# IgD attenuates the IgM-induced anergy response in transitional and mature B cells

**DOI:** 10.1038/ncomms13381

**Published:** 2016-11-10

**Authors:** Zahra Sabouri, Samuel Perotti, Emily Spierings, Peter Humburg, Mehmet Yabas, Hannes Bergmann, Keisuke Horikawa, Carla Roots, Samantha Lambe, Clara Young, T. Dan Andrews, Matthew Field, Anselm Enders, Joanne H. Reed, Christopher C. Goodnow

**Affiliations:** 1 Department of Immunology, John Curtin School of Medical Research, The Australian National University, 131 Garran Rd, Acton, Australian Capital Territory 2601, Australia; 2 Immunology Division, The Garvan Institute of Medical Research, 384 Victoria Street, Darlinghurst, New South Wales 2010, Australia; 3 Department of Genetics and Bioengineering, Trakya University, 22030 Edirne, Turkey; 4 St Vincent's Clinical School, School of Medicine, University of New South Wales, Darlinghurst, New South Wales 2010, Australia

## Abstract

Self-tolerance by clonal anergy of B cells is marked by an increase in IgD and decrease in IgM antigen receptor surface expression, yet the function of IgD on anergic cells is obscure. Here we define the RNA landscape of the *in vivo* anergy response, comprising 220 induced sequences including a core set of 97. Failure to co-express IgD with IgM decreases overall expression of receptors for self-antigen, but paradoxically increases the core anergy response, exemplified by increased *Sdc1* encoding the cell surface marker syndecan-1. IgD expressed on its own is nevertheless competent to induce calcium signalling and the core anergy mRNA response. Syndecan-1 induction correlates with reduction of surface IgM and is exaggerated without surface IgD in many transitional and mature B cells. These results show that IgD attenuates the response to self-antigen in anergic cells and promotes their accumulation. In this way, IgD minimizes tolerance-induced holes in the pre-immune antibody repertoire.

## Abstract (web summary)

Self-reactive B cells that are anergic express mainly IgD, yet the function of IgD is not clear. Here the authors analyse primary B cells from mice to show that IgD signalling attenuates self-antigen induced gene expression and promotes survival of anergic B cells that might go on to reactivate to foreign antigens and mutate away from self-reactivity.

## Graphical abstract

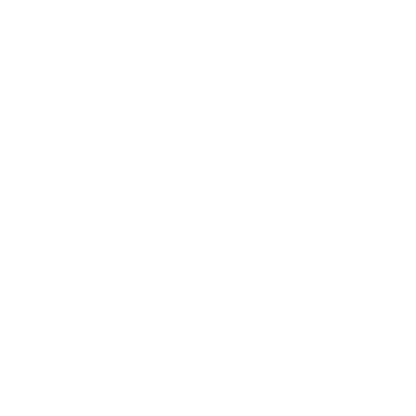

Clonal anergy is an enigmatic mechanism for actively acquired tolerance, a process in which self-reactive cells remain in the lymphocyte repertoire of secondary lymphoid tissues but are deficient in generation of effector progeny^1^,^2^. Anergy is best characterized in mouse and human peripheral B cells expressing high cell surface levels of IgD and low levels of IgM B cell receptors (BCR), which account for 10–50% of the mature pre-immune B cell repertoire, depending on an arbitrary cut-off for low surface IgM (refs 3, 4, 5, 6, 7). Retaining anergic B cells bearing self-binding antibodies in the secondary lymphoid organs presents a risk of autoimmunity^8^, as the diminished proliferation and antibody secretion that characterizes anergic B cells is potentially reversible^2^,^9^. Pathological proliferation of B cells that would normally be anergic also leads to common adult malignancies, exemplified by a large subgroup of chronic lymphocytic leukaemia cases^10^, and by the over-representation of B cells using self-reactive VH4-34 heavy chains, which are normally anergic, within the poor prognosis subset of diffuse large B cell lymphoma^11^. By contrast, physiological proliferation of B cells that were initially anergic has been shown to occur when these cells bind a foreign antigen recognized by T-follicular helper cells and produce germinal centre (GC) progeny and IgG antibodies that have been hypermutated away from self-reactivity^12^,^13^. The molecular nature of B cell anergy that precedes any reactivation into proliferation nevertheless remains unresolved, in particular whether or not anergy is explained by binding antigen primarily through IgD antigen receptors.

Anergic cells selectively inhibit trafficking of nascent IgM but not IgD through the trans-Golgi network to the cell surface^14^. A similar change in IgM trafficking occurs in malignant B cells in chronic lymphocytic leukaemia^15^ and during normal maturation of B cells in the spleen^16^. This altered trafficking may be explained by the IgD juxtamembrane and transmembrane segments—one of the few evolutionarily conserved domains of IgD (ref. 17)—associating preferentially with the CD79αβ subunits needed for IgM and IgD trafficking and signalling on the cell surface^18^,^19^,^20^,^21^. Immature B cells begin by expressing only IgM, but IgD co-expression progressively increases as they become transitional and mature B cells in the spleen due to increased expression of *Zfp318* (ref. 22), which facilitates alternative mRNA splicing of the heavy chain variable (VDJ_H_) exon to either IgM or IgD heavy chain constant (C)-region exons. This arrangement is evolutionarily preserved in most species of fish, amphibians, reptiles, birds and mammals^17^,^23^, yet mice lacking IgD have normal B cell development and only slightly delayed antibody responses^24^,^25^. Likewise, comparison of mice that express only IgM or only IgD reveals no discernable difference in the capacity of these alternative receptors to promote B cell development, tolerance, activation or antibody secretion *in vivo*^26^,^27^.

Conflicting results from tissue culture experiments support the idea that IgM and IgD signal differently. Early experiments suggested that IgM signals drive apoptosis whereas IgD signals induce proliferation, so that immature IgM-only B cells would pass through a window of obligate tolerance susceptibility^28^,^29^. However, these differences might reflect the use of different antibody ligands to engage IgM and IgD, rather than an intrinsic difference in the receptor isotypes. In transfected B lymphoma cells, a single haptenated antigen signalled through IgD more strongly and durably than through IgM^30^. By contrast, hen egg lysozyme (HEL) antigen signalling induced CD86 identically through IgD or IgM on splenic B cells expressing one or other isotype^21^. However, when the same IgM^HEL^ and IgD^HEL^ BCRs were expressed separately in a pro-B cell line with a partially crippled BLNK (SLP65) intracellular signalling adaptor, soluble monovalent HEL antigen signalled an increase in intracellular calcium when it bound IgM but induced no calcium signalling when it bound IgD, whereas multivalent HEL-antigen signalled through both isotypes^31^. Extrapolating the findings from the BLNK mutant cell line, it was concluded that the predominant expression of IgD on anergic cells prevents any response to monovalent self-antigens, ascribing the *in vivo* state of anergy to the change in BCR isotype^31^.

Here we directly address the role of IgD on anergic B cells *in vivo* with three complementary approaches, by analysing anergic B cells in mice either lacking IgD, with a novel point mutation in IgD, or inactivation of the IgD-splicing factor *Zfp318*. We find little intrinsic signalling difference between the two receptor isotypes, but IgD has an essential function when co-expressed with IgM, attenuating the *in vivo* response to self and promoting accumulation of mature anergic B cells to increase their availability to encounter foreign antigens and potentially form GCs.

## Results

### Calcium signalling by IgD and IgM

**Figure 1:**
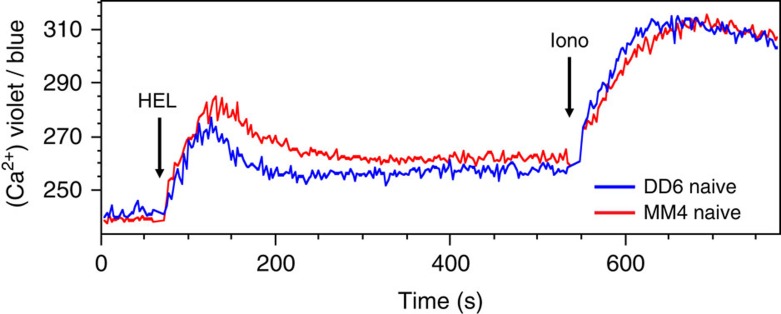
IgD and IgM both signal intracellular calcium upon binding monovalent antigen. Mixtures of splenic naïve B cells from DD6 CD45.2 mice expressing only IgD^HEL^ and MM4 CD45.1 heterozygous mice with only IgM^HEL^ were loaded with the intracellular calcium indicator, Indo-1 and analysed by flow cytometry using gating strategy shown in Supplementary Fig. 1. Arrows indicate when HEL (5 μg ml^−1^) or ionomycin was added. Results are representative of three stimulations and two independent experiments with MM4 and DD6 mice of reversed CD45.1/CD45.2 genotypes.

We first tested the proposal that IgD is unable to trigger an acute elevation of intracellular calcium in response to monomeric antigens like soluble HEL (ref. 31), potentially explaining the unresponsive state of anergic B cells. The intracellular calcium increase elicited by monomeric HEL was directly compared in splenic B cells from MM4 and DD6 transgenic mice, which respectively express the IgM^HEL^ or IgD^HEL^ antigen receptors studied in ref. 31 comprising identical variable regions and different constant regions. In contrast to the findings made in BLNK-mutant pro-B cells^31^, when tested here in mature B cells with normal BLNK both isotypes signalled an acute and sustained calcium response (Fig. 1), although the initial rise in calcium was slightly decreased in cells with IgD^HEL^ antigen receptors.

### IgD enhances formation of GC cells from anergic B cells

**Figure 2:**
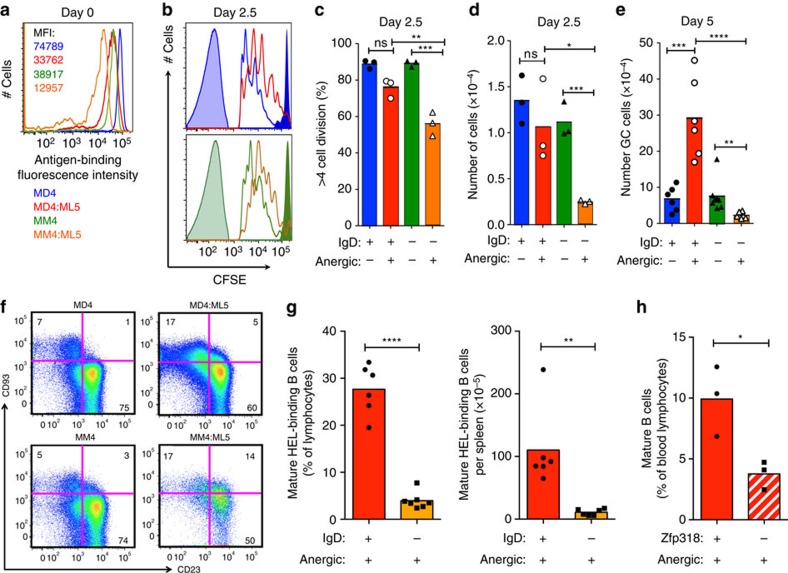
IgD promotes accumulation of mature anergic B cells and germinal centre cell progeny. (**a**–**e**) Adoptive transfer of equal numbers of CFSE-loaded CD45.1^+^ spleen B cells, either naive B cells from MD4 transgenic mice co-expressing IgM^HEL^ and IgD^HEL^ (blue) or MM4 transgenic mice expressing only IgM^HEL^ (green) or anergic B cells from MD4:ML5 (red) or MM4:ML5 (orange) double-transgenic mice. Schematic of experimental strategy shown in Supplementary Fig. 2. Symbols show individual recipients and columns arithmetic means. (**a**) Relative number of HEL-binding antigen receptors per donor B cell before transfer, expressed as geometric mean fluorescence intensity (MFI, arbitrary units). (**b**) CFSE fluorescence histograms 2.5 days after transfer and immunization, using gating strategy in Supplementary Fig. 3. Unshaded histograms show B220^+^CD45.1^+^ HEL-binding donor B cells in the spleen, colour coded as in (**a**). To provide a reference for undiluted CFSE, dark shaded histograms are B220-negative CD45.1^+^ lymphocytes in recipients of MD4 or MM4 cells. A reference for CFSE-negative cells is provided by the lightly shaded histograms from B220^+^CD45.1^−^ recipient B cells. (**c**) Percentage of donor B cells on day 2.5 with CFSE diluted to less than 1/16th of undivided donor cells. (**d**) Number of donor B cells in the spleen on day 2.5. (**e**) Number of Fas^+^CD38^−^CD45.1^+^B220^+^ HEL-binding GC B cells in the spleen 5 days after transfer and immunization, using the gating strategy in Supplementary Figs 4–6. There was no significant difference between the four groups in total GC B cells (CD45.1+ and CD45.1−) as a % of B220+ cells (*P*=0.17; Supplementary Fig. 7). Statistical analysis by ANOVA with Bonferroni's Multiple Comparison post-test: ns=not significant, *P<0.05, ^**^P<0.01, ^***^P<0.001. Representative of two independent experiments. (**f**–**h**) Accumulation of mature anergic B cells with or without IgD in unimmunized double transgenic mice. (**f**) Representative analyses of HEL-binding B220^+^ spleen cells, gated as shown in Supplementary Fig. 8, showing percentage of B cells in indicated gates. (**g**,**h**) Percentage of all lymphocytes that are CD93^−^CD23^+^ HEL-binding B220^+^ anergic B cells in the spleen of individual MD4:ML5 and MM4:ML5 double transgenic mice (**g**) or in the blood of MD4:ML5 double transgenic mice with homozygous null or wild-type *Zfp318* (**h**). Statistical analysis by *t*-test, representative of two independent experiments.

Although IgD expression ceases on activated and GC B cells, we investigated if IgD had any role in the reactivation of anergic B cells using an animal model where anergic B cells have been shown to be reactivated by foreign antigen to form GC progeny, hypermutate their V-regions and rapidly evolve antibodies with lower affinity for self-antigen^12^. HEL-specific anergic B cells were obtained from MD4:ML5 animals that co-express IgD^HEL^ with IgM^HEL^, or from MM4:ML5 animals where the heavy chain transgene lacks the IgD exons and encodes only IgM^HEL^. The mice also carried the ML5 transgene producing circulating HEL at sufficient concentration to induce B cell anergy^1^,^26^. Naïve (non-anergic) control B cells with or without IgD were obtained from MD4 or MM4 single-transgenic mice. After labelling with CFSE dye, equal numbers of splenic B cells bearing the CD45.1 congenic marker were adoptively transferred into C57BL/6 (CD45.2) mice. The recipients were immunized with HEL coupled to sheep red blood cells, the latter eliciting T follicular helper cells to form GCs (HEL-SRBC). At the time of transfer, IgD^+^ anergic B cells had three times more HEL-specific antigen receptors on their surface compared with anergic IgM-only B cells (red versus orange histograms, Fig. 2a).

Half of the recipients were analysed after 2.5 days. The progeny of naïve control B cells had divided and diluted CFSE comparably with or without IgD (Fig. 2b, unshaded blue versus green histograms), 90% diluting by more than 4 cell divisions (Fig. 2c). By comparison, 75% of IgD+ and 55% of IgD-deficient anergic cell progeny had divided >four times (red and orange histograms, Fig. 2b,c). The total number of progeny from IgD-deficient anergic cells was decreased to only 20% of naïve controls by day 2.5 (Fig. 2d), whereas progeny of IgD^+^ anergic cells accumulated to the same number as controls.

The remainder of the recipients were analysed after 5 days. Fas+GL7+GC B cells in the spleen averaged 2.6% of B cells and were not significantly different between the groups of recipients, and CD45.1^+^ HEL-binding donor GC B cells accounted for 9% of GC cells averaged across the groups. However, anergic IgD^+^ B cells formed more GC progeny than naïve IgD^+^ counterparts, as observed previously^12^, but the opposite was observed for anergic B cells without IgD, which formed many fewer GC progeny than their naïve counterparts (orange bar, Fig. 2e). By contrast, IgD-deficiency did not alter the number of GC progeny formed from naïve B cells on day 5 (green bar).

### IgD promotes accumulation of mature anergic B cells

One explanation for the results above was that IgD assists short-lived anergic B cells to survive in the spleen long enough to respond to foreign antigen and T cell help. Consistent with this hypothesis, mature CD93^−^CD23^+^ anergic B cells accumulated to much lower frequency in the spleen of IgD-deficient MM4:ML5 double transgenic animals compared with MD4:ML5 animals (Fig. 2f,g). To exclude differences in transgene integration site between the MD4 and MM4 animals, we also prevented IgD co-expression in MD4:ML5 transgenic mice by breeding with a *Zfp318* null allele^22^. Accumulation of anergic mature B cells was diminished in *Zfp318*^*−/−*^ MD4:ML5 transgenic mice where the B cells only expressed IgM^HEL^ (Fig. 2h).

### IgD promotes accumulation independently of effects on IgM

**Figure 3:**
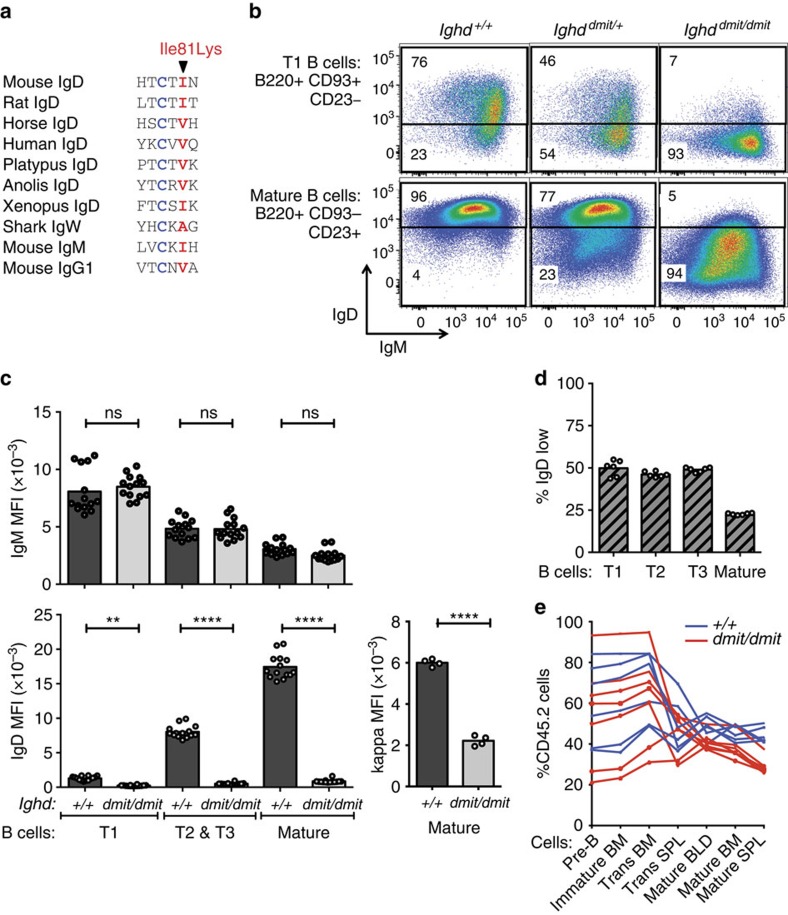
Surface IgD promotes mature B cell accumulation independently of effect on IgM. (**a**) Alignment of β-sheet F in C_H_1 domains of the indicated proteins, showing the hydrophobic core Ile residue mutated to Lys in IgD of the *dimit* strain (red), flanking the Cys forming the intradomain disulphide bond (blue). (**b**) Representative IgD and IgM expression on splenic B cells from mice of the indicated *Ighd* genotypes, gated as shown in Supplementary Fig. 9 on the CD93^+^CD23^−^ T1 subset (top) or the CD93^−^CD23^+^ mature follicular subset (bottom). Shown are gates used to resolve cells expressing the mutant or wild-type *Ighd* alleles in heterozygotes. Note that wild-type IgD is expressed at 10-fold lower MFI on T1 cells, necessitating a lower gate for T1 cells expressing wild-type IgD. (**c**) Surface IgM, IgD and kappa light chain MFI on the indicated B cell subsets from individual mice of the indicated genotypes, and mean for each subset. IgM, IgD data pooled from two separate experiments. Statistical analysis by *t*-test: ns (not significant) *P*>0.05; *P<0.05; **P<0.01; ***P<0.001; ****P<0.0001. (**d**) Percentage of immature, transitional and mature spleen B cells expressing the mutant *Ighd* allele in individual *Ighd*^*dmit/+*^ heterozygous mice. (**e**) Irradiated wild-type CD45.1 recipients received 10^6^ wild-type CD45.1 bone marrow cells mixed with 10^6^ CD45.2 marrow cells from *Ighd*^*dmit/dmit*^ or *Ighd*^*+/+*^ donors. Lines show percentage CD45.2^+^ cells in the indicated B cell subsets in bone marrow, blood and spleen of individual chimeric mice 8 weeks after transplantation, gated as shown in Supplementary Fig. 10. Statistical analysis by three-way ANOVA indicated a significant decrease in the representation of CD45.2 mature cells between the transitional and mature stages of development (*P*<0.001), and a significant effect of IgD on this decrease (*P*=0.005) that did not appear to be different between tissues (*P*=0.099).

Diminished accumulation of mature anergic B cells in the experiments above could reflect either a difference between surface IgM and surface IgD in the way they trigger responses to self-antigen, or a loss of the attenuating effect of IgD on surface IgM levels, or both. In an ENU mutagenesis screen, we discovered a surface IgD-deficient C57BL/6 mouse strain with unaltered levels of surface IgM. The *dimit* strain (C57BL/6JAnu-*Ighd*^*dmit*^/Apb) has acquired a single T>A point mutation resulting in an Ile81Lys substitution within the Cδ1 exon (Fig. 3a). Ile81 flanks Cys79 that forms the intradomain disulphide bond and corresponds to an obligatory hydrophobic residue at position 5 within β-sheet F of all Ig C_H_1 domains, part of a folding nucleus of inward-pointing hydrophobic residues in β-sheets B, C, E and F^32^,^33^. Ile81 mutation to charged, hydrophilic lysine would be expected to prevent folding of the IgD C_H_1 domain into the conformation needed to pair with immunoglobulin light chains.

Surface IgD progressively increased on wild-type spleen B cells between immature (CD93^+^CD23^−^ transitional 1, T1), semi-mature transitional cells (CD93^+^CD23^+^ T2 / T3 subset), and mature follicular cells (CD93^−^ CD23^+^)^34^, but was decreased to less than 5% of normal levels in *Ighd*^*dmit/dmit*^ homozygotes (Fig. 3b,c). The mean surface IgM and the broad cell to cell variance was nevertheless identical in *Ighd*^*dmit/dmit*^ homozygotes and wild-type controls (Fig. 3c). This contrasts with the aberrantly high cell surface IgM on B cells in IgD knockout or *Zfp318* knockout mice^22^,^24^,^25^, where IgD no longer competes with IgM for assembly with CD79αβ (refs 18, 20). The loss of IgD and normal surface IgM caused an overall 65% decrease in mean BCR numbers on mature B cells (Fig. 3c).

The IgD point mutation enabled testing if loss of surface IgD affected mature B cell accumulation independently from IgD's inhibitory effect on surface IgM levels. In heterozygous *Ighd*^*dmit/+*^mice, allelic exclusion resulted in 50% of the immature T1, T2 and T3 B cells expressing the wildtype *Ighd* allele and 50% expressing the mutant allele, the latter distinguished as IgD^low^ cells by flow cytometry (Fig. 3b). By contrast, B cells expressing the surface IgD-deficient allele contributed poorly to the mature B cell repertoire and accounted for only 24% of cells (Fig. 3d). A similar requirement for surface IgD for the accumulation of mature B cells relative to immature B cells was observed in the bone marrow, blood and spleen of mixed bone marrow chimeras reconstituted with a mixture of CD45.1 and CD45.2 bone marrow (Fig. 3e). The 52% decrease in mature B cells with the *Ighd*^*dmit*^ mutation appears less than the decrease in mature anergic B cells in MD4:ML5 transgenic mice with the *Zfp318* mutation or the decrease in MM4:ML5 anergic cells lacking IgD. This may reflect the fact that the latter two lose two functions of IgD: surface IgD display and IgD attenuation of surface IgM expression, whereas the *Ighd*^*dmit*^ mutation selectively disrupts surface IgD.

### IgD attenuates anergic B cell mRNA response to self-antigen

We next performed a global RNA analysis of MD4 and MM4 naïve and anergic mature B cells to test for any evidence that surface IgD and IgM differ in their capacity to elicit responses to self-antigen. Two variables differ between MD4:ML5 and MM4:ML5 anergic B cells: (1) surface IgM is lower on MD4:ML5 cells because IgD competes with IgM for CD79 assembly and surface expression; (2) the total cell surface pool of antigen-binding receptors is higher on MD4:ML5 cells, because IgD is not downregulated on anergic cells. The null hypothesis, that there is no difference in the response to self elicited by surface IgM and IgD, would therefore predict that there should be no difference in the induction of anergy RNAs or there should be *less* induction of some or all anergy RNAs in MM4:ML5 cells because of their 65% fewer receptors for self-antigen. The opposite result—a higher response to self by some or all of the anergy-induced RNAs in MM4:ML5 cells without IgD—would demonstrate that IgM is better at eliciting that part of the response than IgD, and since IgD decreases surface IgM that could further exaggerate any difference in the activity of the surface receptors themselves. Much higher induction of all anergy genes in MM4:ML5 cells, and little or no response to self-antigen in MD4:ML5 cells, would be predicted based on the hypothesis that IgD cannot signal to monomeric HEL (ref. 31).

**Figure 4:**
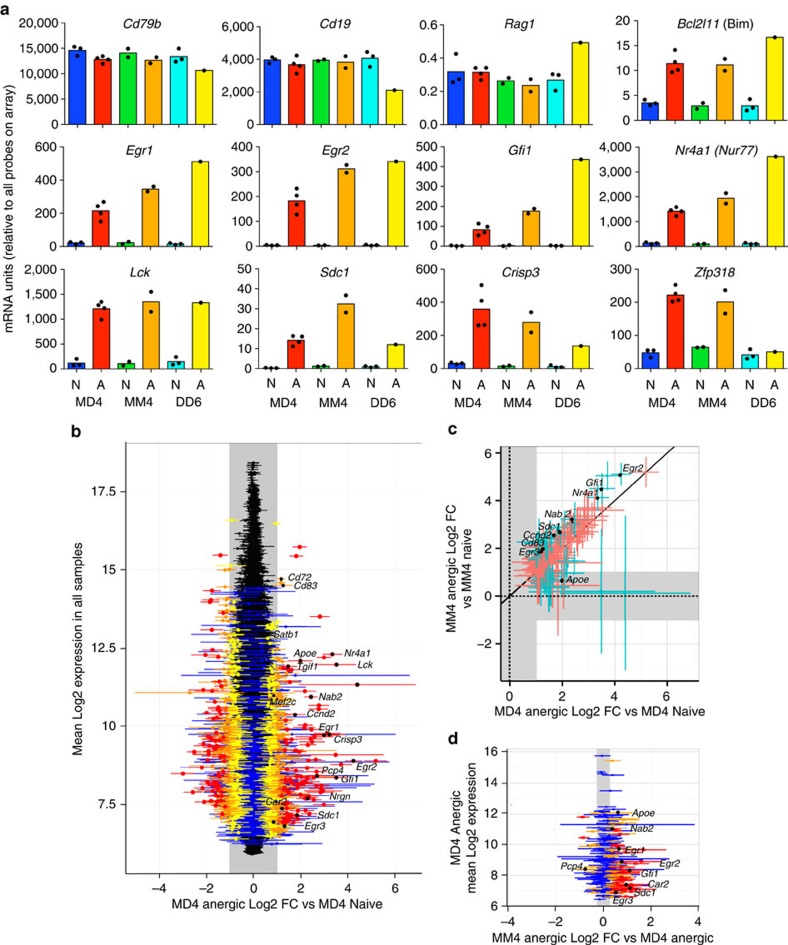
Impact of IgD-deficiency on the anergic B cell gene expression program. Naïve (N) or anergic (A) HEL-binding CD93^−^CD23^+^ mature spleen B cells were sorted from individual MD4 or MD4:ML5 (co-expressing IgM^HEL^ and IgD^HEL^), MM4 or MM4:ML5 (only expressing IgM^HEL^), and DD6 or DD6:ML5 (only expressing IgD^HEL^) transgenic mice, and mRNA analysed on Agilent microarrays. (**a**) Analysis by Agilent feature extraction software. Dots show values and columns arithmetic means for indicated mRNA probes from independent mice per genotype, except DD6:ML5 where RNA was pooled from three sorted donors. (**b**–**d**) Analysis by limma. (**b**) *Y*-axis, mean expression in all samples for 33,653 mRNA probes with positive expression in anergic B cells. *X*-axis, mean and 95% confidence interval (CI) for ≥2-fold change (FC, grey shaded region) in MD4:ML5 anergic cells relative to MD4 naïve cells: red, strong evidence; orange, moderate evidence; yellow, weak evidence; blue, very weak; black, no evidence. Black squares and gene symbols show mean FC for previously identified anergy-induced mRNAs. (**c**) 220 probes with strong or moderate evidence of ≥2-fold increase in MD4:ML5 anergic cells. *X*-axis as in (**b**). *Y*-axis, mean and 95% CI of ≥2 FC in expression in MM4:ML5 anergic cells relative to MM4 naïve cells. (**d**) 220 anergy-induced probes in (**c**), showing mean and 95% CI of ≥1.25 FC in MM4:ML5 anergic cells without IgD relative to MD4:ML5 anergic cells with IgD. Black squares and accompanying gene symbols are previously identified anergy-induced mRNAs with differential expression in IgD-deficient anergic cells.

Anergic and naïve mature CD93^−^CD23^+^ HEL-binding splenic B cells were sorted from MD4:ML5 or MM4:ML5 mice and from MD4 or MM4 single-transgenic controls. As an additional comparison, mature HEL-binding B cells that expressed only IgD^HEL^ were sorted from DD6:ML5 or DD6 transgenic mice, which carry the same transgene as MD4 but missing the IgM exons^26^. RNA from independent donors was measured on Agilent microarrays carrying 59,305 different oligonucleotide probes for protein-coding and non-coding RNAs (Supplementary Data 1). Analysis of constitutively expressed mRNAs in B cells, exemplified by *Cd19, Cd79b, B2m* and *Gapdh*, showed comparable expression regardless of BCR isotype or exposure to self-antigen (Fig. 4a and Supplementary Fig. 11a).

In B cells that lose surface Ig completely, tonic BCR signals through PI3-kinase are lost as evidenced by an increase in mRNAs for four PI3-kinase repressed genes, *Rag1*, *Cdkn1b, Bcl2l11* and *Aicda* (ref. 35). Hence it was conceivable that the 65% decrease in surface BCRs on anergic MM4:ML5 cells compared with anergic MD4:ML5 cells or naïve MM4 cells (Fig. 2a) might also increase these mRNAs, but there was no evidence that this was the case (Fig. 4a and Supplementary Fig. 11b).

We next analysed 18 mRNAs previously identified as being consistently induced in anergic MD4:ML5 B cells using first-generation Affymetrix arrays^36^. Twelve of these mRNAs (*Egr1, Egr2, Gfi1, Nab2, Cd83, Tgif1, Lck, Cd72, Nrgn, Ccnd2, Crisp3* and *Pcp4*) were consistently increased in anergic B cells relative to naïve B cells, regardless of whether the B cells co-expressed IgM and IgD or only expressed one isotype (Fig. 4a and Supplementary Fig. 11c). Despite 62% fewer surface antigen receptors on IgD-deficient anergic cells, *Egr1, Egr2*, *Gfi1* and *Nab2* were paradoxically induced to 1.25–2.3 fold higher levels in IgD-deficient MM4:ML5 anergic cells compared with IgD+ MD4:ML5 anergic cells. The *Egr2* paralogue, *Egr3*, and two other genes shown previously to be induced in anergic B cells, *Nr4a1* (NUR77(ref. 7)) and *Sdc1* (CD138 (refs 37, 38)), were also induced to higher levels in MM4:ML5 IgD-deficient anergic B cells (Fig. 4a and Supplementary Fig. 11d).

To obtain a comprehensive transcriptional landscape of B cell anergy with and without IgD, we analysed all 59,305 oligonucleotide probes on the arrays. Using limma^39^, we focussed on 33,653 probes that were positive for expression in anergic B cells (Supplementary Data 2). Of these, 97 probes had strong evidence of at least two-fold increased expression in MD4:ML5 anergic B cells compared with MD4 naïve counterparts, including 13 previously described anergy genes (Table 1 and Fig. 4b). In this set of 97 probes, 91 and 78% had moderate or strong evidence of induction in MM4:ML5 and DD6:ML5 anergic B cells, respectively, compared with only 1 and 3% of all 33,653 expressed probes (Fig. 4c and Table 1). An additional 123 probes had moderate evidence of induction in MD4:ML5, and the majority were also induced in anergic B cells with only IgM or only IgD (Table 1). Thus, when chronically stimulated by monomeric self-HEL antigen *in vivo*, IgD expressed with IgM or on its own induces the great majority of the transcriptional response, in contrast to recent conclusions from a BLNK-deficient pro-B cell line^31^.

Of 97 and 123 probes with strong and moderate evidence, respectively, of induction in anergic cells co-expressing IgD and IgM, 34 and 29% had moderate evidence of ≥125% increased expression in MM4:ML5 anergic cells lacking IgD compared with MD4:ML5 anergic cells with IgD (Fig. 4d, Table 1). By contrast, only 4% of the 33,653 expressed probes were increased ≥125% in MM4:ML5 samples. The hyper-induced gene set in IgD-deficient cells included mRNAs encoding transcription regulators (*Egr1, Egr2, Egr3, Gfi1, Lef1, Ahr, Myb, Myc, Nr4a1, Sox4, Hmgn3, Apex1*), apoptosis inducers (*Casp4*), and cell surface proteins (*Sdc1*, *Il18r1, Dlk1*). Reciprocally, there was no evidence for diminution of a subset of the anergy response without IgD: only 5% of anergy induced genes were decreased without IgD, which is comparable to 4% of all expressed probes on the array showing similar evidence for decreased expression (Table 1). Thus, IgD was not required for induction of the anergy mRNA program but, paradoxically, its co-expression increased the number of self-antigen binding receptors nearly 3-fold (Fig. 2a) yet attenuated one third of the mRNA response to self-antigen.

### Hyper-induction of EGR1, EGR2 and SDC1 proteins

**Figure 5:**
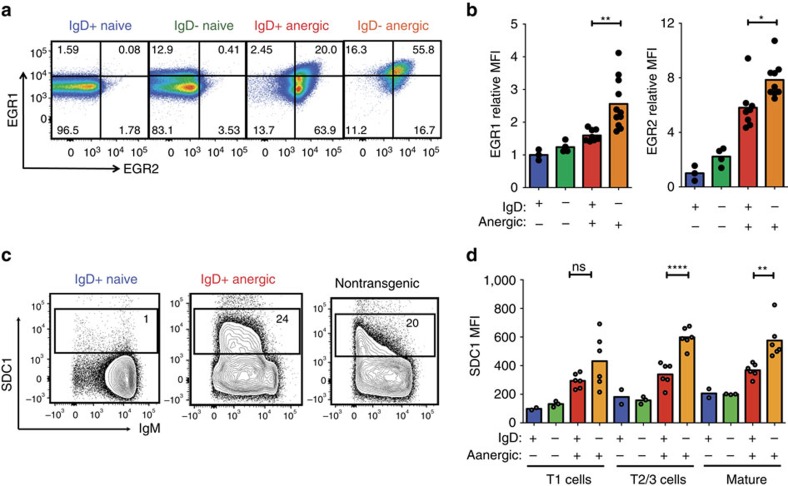
Increased EGR1, EGR2 and SDC1 induction on anergic B cells lacking IgD. Flow cytometric analysis of HEL-binding B220^+^ spleen B cells from MD4 and MM4 transgenic mice (IgD^+^ and IgD^−^ naïve, respectively) and from MD4:ML5 and MM4:ML5 double transgenic mice (IgD+ and IgD^−^ anergic, respectively), gated as shown in Supplementary Figs 12 and 13. (**a**) Representative staining of permeabilised cells for EGR1 and EGR2, showing % HEL-binding B220^+^ cells within the indicated quadrants. (**b**) EGR1 or EGR2 MFI in cells from individual mice pooled from two experiments, normalized to mean MFI in MD4 B cells from the same experiment. (**c**) Representative staining for surface SDC1 and % within the SDC1^+^ gate (set using fluorescence minus one control) for IgD^high^B220^+^ spleen cells from MD4, MD4:ML5 and C57BL/6 mice. (**d**) SDC1 MFI on HEL-binding B220^+^ T1, T2/3 and mature follicular B cell subsets gated as in Fig. 1f. Dots show data from individual mice pooled from two experiments, and columns show means. Statistical analysis by ANOVA with Sidak's multiple comparisons test of selected pairs: ns, not significant; ****P<0.0001; **P<0.01; *P<0.05.

We validated the observations above by antibody staining and flow cytometry, focussing on SDC1 because it is a cell surface protein induced in B cells with chronically elevated intracellular calcium^38^, and on the transcription factors EGR1 and EGR2, which are induced in anergic B cells by the ERK and calcium-calcineurin-NFAT signalling pathways, respectively^4^,^36^,^40^. Anergic B cells from MD4:ML5 animals had significantly increased EGR1 and EGR2 intracellular staining compared with naïve MD4 B cells, which served as a negative control B cell population because they have very low *Egr1* and *Egr2* mRNA, (Fig. 5a,b). Consistent with the microarray findings, there was exaggerated induction of both proteins in IgD-deficient anergic cells from MM4:ML5 animals (Fig. 5b, orange versus red columns).

Cell surface staining confirmed that SDC1 was specifically induced on immature, transitional and mature anergic B cells from MD4:ML5 animals compared with naïve MD4 B cells (Fig. 5c,d, red versus blue columns), and was induced to higher levels on IgD-deficient anergic B cells from MM4:ML5 mice (Fig. 5d, orange columns). A similar level of SDC1 was present on IgM^low^ splenic B cells in C57BL/6 mice with a diverse antibody repertoire (Fig. 5c), consistent with evidence that surface IgM downregulation identifies anergic self-reactive B cells in the normal repertoire^3^,^4^,^5^,^6^,^7^.

### IgD attenuates SDC1 induction independently of effect on IgM

**Figure 6:**
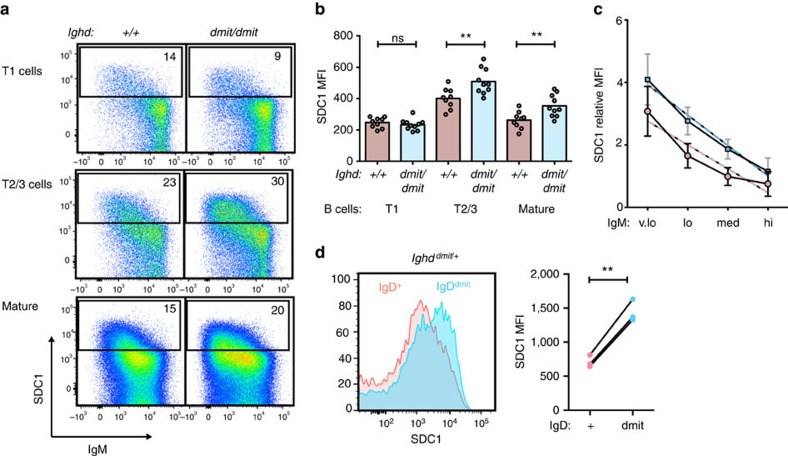
IgD attenuates SDC1 induction independently of its effect on surface IgM. (**a**–**c**) Flow cytometric analysis of spleen cells from *Ighd*^*+/+*^ and *Ighd*^*dmit/dmit*^ mice. (**a**) Representative plots gated on the indicated subsets of B220+ cells, and %SDC1^+^ cells. (**b**) SDC1 MFI on the indicated B cell subsets in individual mice pooled from three independent experiments. Columns, arithmetic means. Statistical comparison by Mann–Whitney test: **P<0.01. (**c**) B220^+^ cells were gated into four equal quartiles of surface IgM fluorescence: very low (v.lo), low (lo), medium (med) and high (hi), as shown in Supplementary Fig. 14. Shown for each quartile is the mean and s.d. SDC1 MFI, expressed relative to the MFI of B cells in the same experiment from a fluorescence minus one control sample where the SDC1 antibody was omitted. Data from *n*=7 *Ighd*^*+/+*^ (red) and *n*=7 *Ighd*^*dmit/dmit*^ (blue) mice pooled from two experiments. Dashed lines are best fit by linear regression analysis. Statistical comparison of linear regression: slopes, ns; intercepts, *P*<0.0001. (**d**) Histograms of SDC1 fluorescence on T3 (IgM^low^CD93^+^CD23^+^) spleen B cells from heterozygous *Ighd*^*dmit/+*^ mice, gated as shown in Supplementary Fig. 9, on IgD^high^ cells expressing the wild-type *Ighd* allele (red), and on IgD^low^ cells expressing the mutant *Ighd* allele (blue). Graph shows SDC1 MFI on *Ighd*^*dmit*^ and *Ighd*^*+*^ T3 B cells within the same individual mouse connected by lines. Statistical analysis by paired *t*-test: **P<0.01. Representative of two independent experiments and *n*=8 animals.

To extend these observations to the normal B cell repertoire, we focussed on SDC1 because cell surface antibody staining could readily detect its induction on individual B cells above background autofluorescence. The increase in SDC1 and decrease in surface IgM varied from cell to cell in a highly correlated way in the immature T1, transitional T2/3, and mature follicular B cell subsets (Fig. 6a). While surface IgM was unaltered in B cells from homozygous *Ighd*^*dmit*^ mice, SDC1 was induced to higher mean levels on the T2/3 and mature B cell subsets with little surface IgD compared with wild-type controls with normal surface IgD (Fig. 6b). When mature B cells were separated into four quartiles based on the amount of surface IgM, mean SDC1 was higher on surface IgD-deficient cells in each quartile (Fig. 6c). SDC1 levels were also analysed in heterozygous *Ighd*^*dmit/+*^ mice, where allelic exclusion enabled comparison within individual animals between the 50% of transitional IgM^low^ T3 B cells expressing the mutant *Ighd* allele and the 50% with wild-type *Ighd*. SDC1 was induced to higher levels on transitional B cells lacking surface IgD (Fig. 6d). Thus surface IgD acts cell-autonomously to attenuate induction of the SDC1 anergy marker on many semi-mature and mature B cells in the normal repertoire, independently of IgD's inhibitory effect on surface IgM levels.

## Discussion

The experiments above advance understanding of self-tolerance and the role of IgD in six ways. First, they define 220 induced RNA species that provide a comprehensive definition of the anergic state *in vivo*, enabling future studies of how anergy is distorted by susceptibility genes in autoimmunity or by mutations in chronic lymphocytic leukaemia^10^ or lymphoma^11^. Second, the global RNA analysis reveals that IgD differs from IgM by attenuating a large subset of the anergy response to self-antigen. Third, the results of the RNA analysis and of calcium signalling did not support previous conclusions that IgD signals for B cell activation while IgM signals for tolerance^28^,^29^, that IgD signals in a stronger and more sustained way than IgM (ref. 30), nor that IgD is unable to signal to monovalent ligands^31^. Fourth, flow cytometric staining for SDC1 reinforces the proposal that IgM downregulation on many B cells in the normal repertoire reflects an active anergic response to self^3^ and yields a method to analyse and sort anergic B cells without engaging surface IgM with antibodies^3^,^4^ nor requiring crosses to a transgenic *Nr4a1:GFP* reporter strain^7^. Fifth, we discover a new mouse mutant that eliminates surface IgD but retains the inhibitory effect of IgD on surface IgM display, and show that the selective loss of surface IgD results in exaggerated induction of SDC1 on IgM^low^ anergic B cells. Finally, the experiments reveal that IgD promotes accumulation of anergic B cells as mature follicular B cells, thus minimizing tolerance-induced holes in the pre-immune repertoire of antibodies available in secondary lymphoid tissues.

Loss of surface IgD on transitional IgM^low^ T3 B cells decreases the number of surface BCRs and would be expected to diminish their response to self-antigen if IgM and IgD were functionally equivalent, but paradoxically it increased the anergy response reported by SDC1. Plasma cells with high levels of SDC1 are absent in *Prdm1* (Blimp-1) knockout mice but the subset of SDC1^low^ mature B cells analysed here are not plasma cells because they form independently of Blimp-1 (ref. 41) and have high B220 and CD19 that are both downregulated on plasma cells. SDC1 is also induced on self-reactive B cells binding the Smith ribonucleoprotein^37^. While MD4 B cells did not express SDC1 in the absence of self-HEL (ref. 37), SDC1 was spontaneously induced on MD4 naïve B cells when their intracellular calcium was chronically elevated by genetic deficiency of inositol 1,4,5-trisphosphate 3-kinase b (*Itpkb*)^38^. Repeated cycles of self-antigen binding in anergic cells raise cytosolic calcium to 100–400 nM in an oscillatory manner, and calcium reverts to the 50 nM baseline of naïve B cells when anergic cells are transplanted into recipients lacking the self-antigen^40^ or when the self-antigen is outcompeted by a non-stimulatory ligand for the BCR (ref. 42). Consistent with the range of SDC1 induction between B cells in non-transgenic mice and its correlation with surface IgM downregulation, intracellular calcium is elevated to varying extents among polyclonal B cells in proportion to their expression of an *Nr4a1:GFP* reporter transgene^7^. Thus SDC1 serves as a convenient reporter for the varying degrees of self-reactivity among B cells in the pre-immune repertoire.

The attenuated self-antigen response when IgD is coexpressed with IgM *in vivo* helps clarify earlier conflicting findings. A small decrease in the proportion of mature B cells was observed comparing a pair of transgenic strains expressing a polyreactive BCR as IgM-only or as IgM and IgD (ref. 43). However, another pair of transgenic mice with a slightly different H-chain V-region displayed more mature B cells when bearing IgM-only than with IgM+IgD, although in these animals transitional IgM-only B cells appeared more susceptible to deletion when exposed to a tolerizing injection of human IgG antigen^44^. Mature B cells were depleted by a tolerizing injection of TNP-dextran in IgM-only Ig^SP6^ transgenic mice, but not when these mice were crossed with an IgD-only Ig^SP6^ transgenic strain so that IgD was co-expressed with IgM (ref. 45). From these results it was hypothesized that IgD attenuates tolerogenic signalling by IgM (ref. 46), consistent with the findings here. Interpretation of these effects was confounded by the absolute requirement for endogenous light chain specificities for Ig^SP6^ B cells to mature, and by rescue of their maturation by injection of TNP-ficoll^47^.

A key question is how much the attenuation of the anergy response by IgD demonstrated here reflects an intrinsic decrease in the ability of IgD to signal in response to self-antigens and how much reflects IgD inhibition of surface IgM for assembly with CD79αβ and trafficking to the cell surface^18^,^19^,^20^,^21^. The experiments with the IgD *dmit* point mutant in Figs 3 and 6 show that exaggerated response to self-antigen still occurs when IgD attenuates surface IgM normally but IgD is no longer expressed on the surface of anergic cells. Thus IgD attenuates the response to self-antigen in two ways: IgD is less able to elicit a subset of the response than IgM on the cell surface, and IgD decreases the display of surface IgM.

A simple explanation for the decreased signalling by IgD on the surface has recently been proposed based on findings that IgD was unable to signal an acute calcium response by monovalent but not multivalent HEL antigen in a pro-B cell line where the BLNK cytoplasmic signalling adaptor was expressed as an oestrogen receptor-chimera that partially crippled BLNK function by binding to heat shock proteins^31^. Four lines of evidence here indicate this black and white difference between IgD and IgM does not extrapolate to primary B cells with normal BLNK. First, in primary B cells IgD was capable of inducing an acute calcium response to monomeric HEL *in vitro* (Fig. 1). The initial calcium peak was consistently slightly lower in IgD^HEL^ B cells than in IgM^HEL^ B cells, and conceivably this subtle difference may be exaggerated in cells with defective BLNK. Secondly, IgD was competent to induce 78% of the anergy-induced RNA species in response to monomeric HEL *in vivo*, including calcium-induced genes like *Egr2*. Third, anergic IgM^low^ B cells in mice with a normal antibody repertoire make antibodies binding to DNA and other polymeric self-antigens^4^,^6^,^7^, making it unlikely that IgD attenuation of SDC1 on a large fraction of these cells reflects recognition of only monovalent self-antigens. Instead, IgD appears to acquire the capacity to attenuate the response when it is co-expressed with IgM. This might reflect competition between the two receptor isotypes for particular signalling molecules or membrane domains^18^,^19^,^21^,^48^,^49^.

The need for IgD to promote anergic B cell accumulation in the spleen and blood may represent a mechanism to minimize holes in the antibody repertoire created by self-tolerance. Deletion of self-reactive B cells in the bone marrow, either by apoptosis or receptor editing, removes antibody specificities from the repertoire before they can be tested for binding to foreign antigens. By contrast, antibodies on anergic B cells can be hypermutated in germinal centres and selected for variants that have lost binding to self but retain binding to foreign microbes^13^.

## Methods

### Mice

All mice were on a C57BL/6 background, male, mean age 157 days (range 84–287 d), maintained in specific pathogen free conditions under a protocol approved and monitored by the ANU Animal Experimentation and Ethics Committee. MD4:ML5, MM4:ML5 and DD6:ML5 transgenic mice were as described^1^,^3^,^26^. The *Ighd*^*dmit*^ strain was isolated by screening ENU-mutagenised C57BL/6 mice for low surface IgD on blood B cells. The ENU-induced mutation was identified by exome sequencing using the SureSelect Mouse Exome kit (G7550A-001: Agilent, CA) and 100 bp paired-end sequencing on an Illumina HySeq2500 following the manufacturers' protocols. Allele-specific genotyping in C57BL/6 backcrossed progeny was performed using Amplifluor assays (Chemicon, Temecula, CA) and primers designed using the Assay architect online tool (http://apps.serologicals.com/AAA/mainmenu.aspx; sequences available on request). Zfp318 knockout mice were as described^22^. For ethical purposes, each experiment used the minimum number of animals per group needed to provide statistical power to detect a two-fold difference between groups, and each experiment was performed multiple times. Animals from the same parents were randomly assigned to experimental groups based on their genotype, but were not blinded to the investigator. No animals were excluded from analysis.

### Flow cytometry and sorting

Preparation of spleen cells and staining for immature and mature B cells as described previously^12^,^22^, employing Efluor 780 fixable viability dye (eBioscience) and the following antibodies: B220, RA3-6B2 coupled to Alexafluor700 or APC-Cy7 (Becton Dickinson, 1/200); Fas, Jo-2 coupled to PE (Becton Dickinson), GL7 coupled to FITC (Becton Dickinson); CD38, clone 90 coupled to PE (Biolegend), CD45.1, A20 coupled to Pacific Blue or Alexfluor700 (Biolegend); CD45.2, 104 coupled to PerCP (Pharmingen); CD93, AA4.1 coupled to FITC, APC or biotin (eBioscience, 1/100) or PE (Biolegend); CD23, B3B4 coupled to PE (BD), PE-Cy7 (eBioscience, 1/200), or Pacific Blue (Biolegend); IgD, 11-26c coupled to FITC or biotin (eBioscience, 1/100), PE (Southern Biotech), PerCP-Cy5.5 or BV510 (Biolegend, 1/200); IgM, II/41 coupled to APC or FITC (BD) or PE-Cy7 (eBioscience, 1/100); Streptavidin coupled to PE-Cy7 or Qdot 605 (eBioscience). Each antibody conjugate and lot was titrated and used at dilutions yielding saturated staining.

In Ig-transgenic mice, edited B cells were eliminated by gating out surface IgKappa^hi^ HEL^low^ B cells after staining with HEL followed by Hy9-APC (1/800) and then anti-kappa monoclonal 187.1 conjugated to PerCP-Cy5.5 (Becton Dickinson, 1/100). Staining for SDC1 employed biotinylated monoclonal antibody 281–2 followed by streptavidin-brilliant violet 605 (Biolegend). EGR1 and EGR2 staining was performed on fixed and permeabilised cells using the Foxp3 staining kit (eBioscience), using rabbit monoclonal antibody T.126.1 (Thermo Scientific) followed by anti-rabbit IgG CF405M polyclonal antibody (Sigma Aldrich) and Erongr2-Phycoerythrin (eBioscience). CFSE labelling, adoptive transfer and immunization with HEL-SRBC was as described^12^.

### Microarray analysis

For microarray analysis, aliquots comprising 10^5^ selected cells were sorted into 15 ml Falcon tubes containing 2 ml foetal calf serum, on ice. The cells were washed in PBS, resuspended in 1,400 μl PBS, transferred to 1.5 ml Eppendorf tubes, centrifuged at 900 g for 6 min at 4 °C, supernatants removed, and tubes with cell pellets snap frozen in liquid N2, stored at −80 °C, and all tubes shipped together on dry ice to Miltenyi Biotec, Germany for processing and microarray hybridization as a single batch.

Cell pellets were lysed and RNA isolated on NucleoSpin RNA II kits (Macherey-Nagel) using standard protocols. RNA quality was analysed on an Agilent 2,100 Bioanalyzer and samples with RIN >6.4 were used. Cy3 labelled cRNA was produced by linear T7-based amplification using the Agilent Low Input Quick Amp Labelling Kit following the manufacturer's protocol, and yields measured with a Nanodrop ND-1,000 Spectrophotometer. 600 ng Cy3-labelled fragmented cRNA was hybridized for 17 h at 65 °C to Agilent Whole Mouse Genome Oligo Microarrays 8 × 60 K according to the Agilent 60-mer microarray processing protocol. After washing, the arrays were scanned on an Agilent Microarray Scanner System. The image files were analysed by two independent methods, Agilent Feature Extraction Software and limma, with similar results. Using Agilent Feature Extraction Software, signal intensities from individual features were normalized by dividing by the median signal of all features on the same array, yielding the relative mRNA units shown for selected probes in Fig. 1a and Supplementary Fig. 11, and for all probes in Supplementary Data 1.

Using limma^39^ as detailed in Supplementary Methods, probe intensities were corrected for background fluorescence using the normal-exponential convolution model implemented by limma followed by cyclic loess normalization. Two low quality samples were excluded from further limma analysis and from Agilent analysis: one of three MM4 naïve and one of three MM4:ML5 anergic samples. To focus on B cell expressed genes, probes with a positive feature score in all MD4:ML5 or MM4:ML5 samples were selected, yielding 33,653 anergic B cell expressed probes (Supplementary Data 2). These were analysed for differential expression between anergic and naïve cells, or between anergic cells with and without IgD. The estimated log_2_ fold change and 95% confidence interval (CI) were computed. A minimum threshold was set for differential expression, with 2-fold chosen for comparison of anergic and naïve B cells, and 1.25-fold for comparing anergic cells with or without IgD. Probes with CIs that overlapped this threshold but had an estimated log fold change beyond the threshold were classed as having moderate evidence of differential expression. Probes where the entire CI fell outside the threshold were classed as having strong evidence of differential expression.

### Data availability

Microarray data that support the findings of this study have been deposited in GEO with the primary accession code GSE82091. All other data that support the findings of this study are available from the corresponding author on request.

## Additional information

**How to cite this article:** Sabouri, Z. *et al*. IgD attenuates the IgM-induced anergy response in transitional and mature B cells. *Nat. Commun.* **7,** 13381 doi: 10.1038/ncomms13381 (2016).

**Publisher's note:** Springer Nature remains neutral with regard to jurisdictional claims in published maps and institutional affiliations.

## Supplementary Material

Supplementary Information Supplementary Figures 1-14

Supplementary Data 1 Microarray data Agilent analysis.

Supplementary Data 2 Microarray data limma analysis.

Peer Review File

## Figures and Tables

**Table 1:** Summary of microarray data analysed by limma.

	**Increased in MD4:ML5 anergic relative to MD4 naive, strong evidence**	**Increased in MD4:ML5 anergic relative to MD4 naive, moderate evidence**	**All expressed genes**
Number unique probes	97	123	33,653
Increased in MM4:ML5 anergic relative to MM4 naive, mod or strong evidence	91%	76%	1%
Increased in DD6:ML5 anergic relative to DD6 naive, mod or strong evidence	78%	61%	3%
Increased in MM4:ML5 anergic relative to MD4:ML5 anergic, mod or strong evidence	34%	29%	4%
Decreased in MM4:ML5 anergic relative to MD4:ML5 anergic, mod or strong evidence	5%	5%	4%
Gene Symbols: Increased in MM4:ML5 anergic relative to MD4:ML5 anergic	*Myb, Gfi1, Egr2, NAP111439-1, Fabp5, Sox4, Lef1, NAP114472-1, Endou, Shmt1, Tacc2, Myc, Rasgef1a, Ms4a4a, Lzts2, Sdc1, Faah, Egr1, Nab2, Ahr, Cenpv, Renbp, Rapgef3, Nefh, Il4i1, LOC100040974, Ppnr, Usp28, 9430078K24Rik, Gad1, Apoe*	*chr17:35080914-35090009_R, 1110038B12Rik, chr8:129336539-129400338_R, Tagap, Tagap1, Egr3, Scn4a, ENSMUST00000103589, Ank, Il18r1, Fam160a1, Echdc3, Car2, Psat1, Eno2, Hmgn3, Slc43a3, Apex1, Casp4, Dhrs3, Vmp1, Slc25a33, Chst7, Cldn10, Ppfibp2, Mcoln2, Prodh, Dlk1, Ubash3b, A_65_P05803*	

Data for all 33,653 probes is provided in Supplementary Table 2.
