# Supplementary material for: IgD attenuates the IgM-induced anergy response in transitional and mature B cells
Source: Nat Commun. 2016 Nov 10;7:13381. doi: 10.1038/ncomms13381 (PMC5109548; doi:10.1038/ncomms13381)
Supplement: Supplementary Information — Supplementary Figures 1-14 [file ncomms13381-s1.pdf]

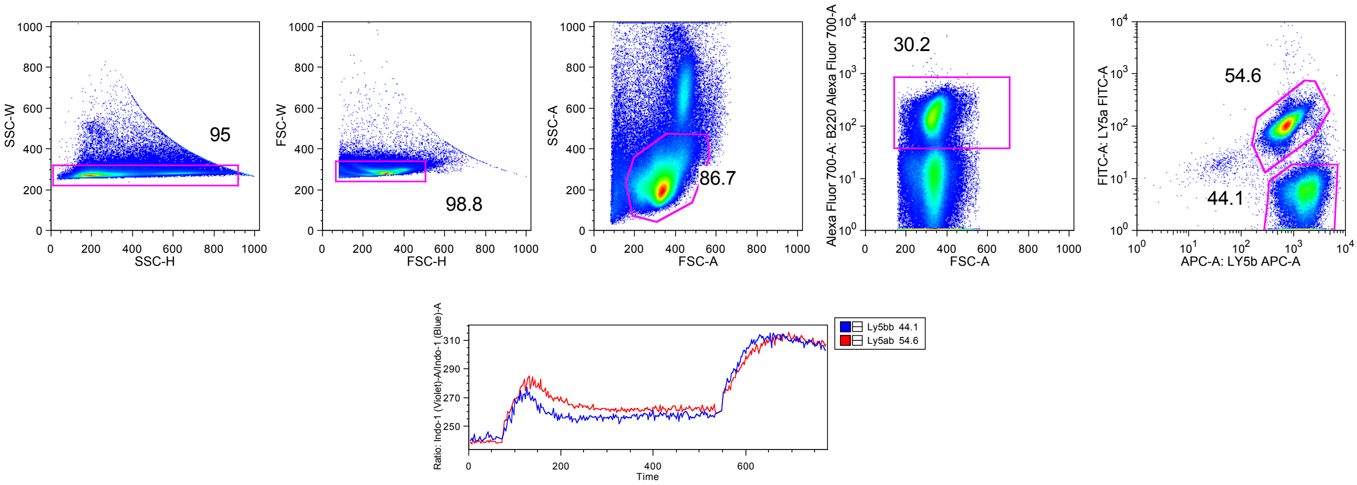

Supplementary Figure 1. Gating Strategy for Figure 1

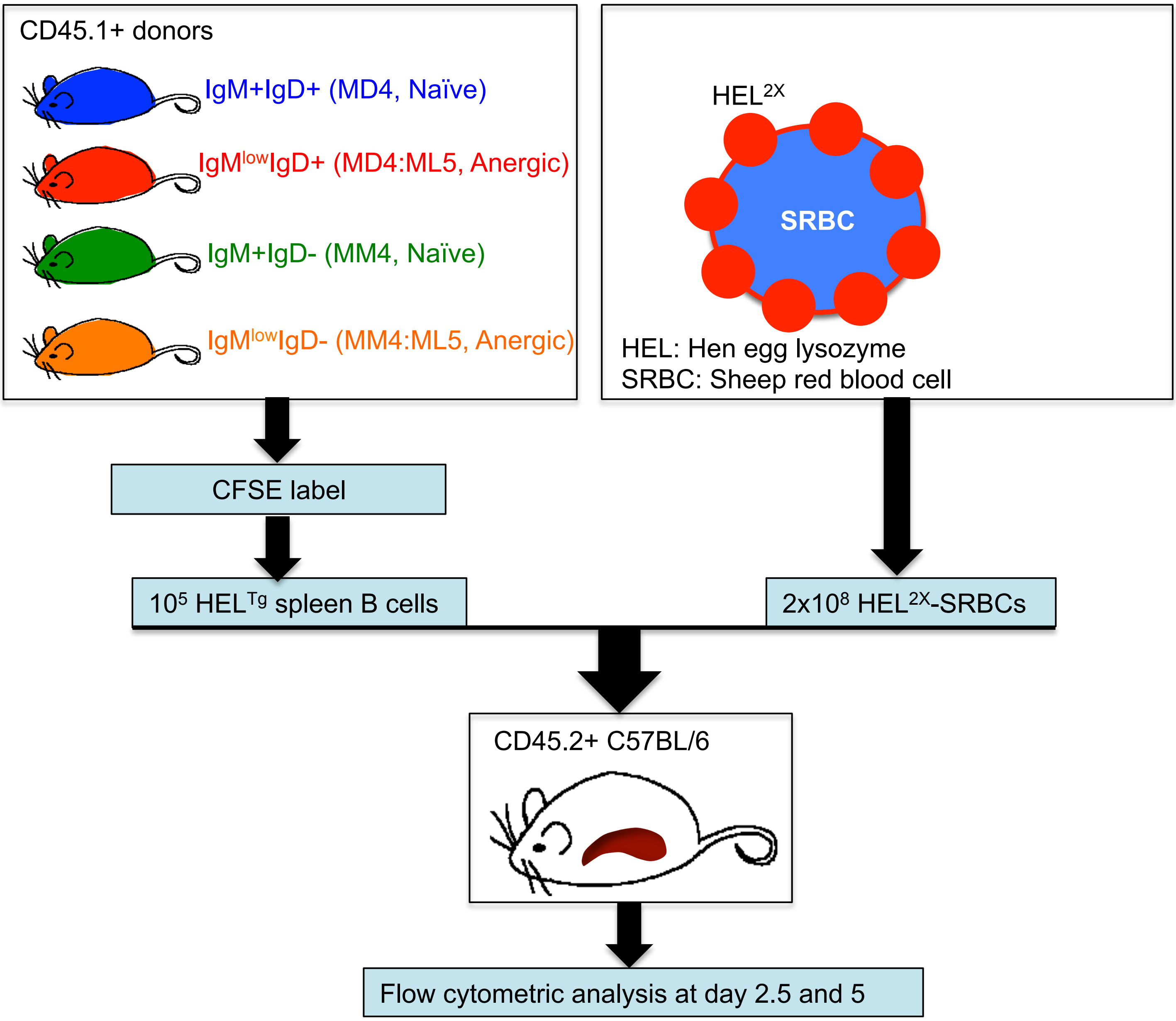

Supplementary Figure 2. Experimental design for Figure 2a-e.

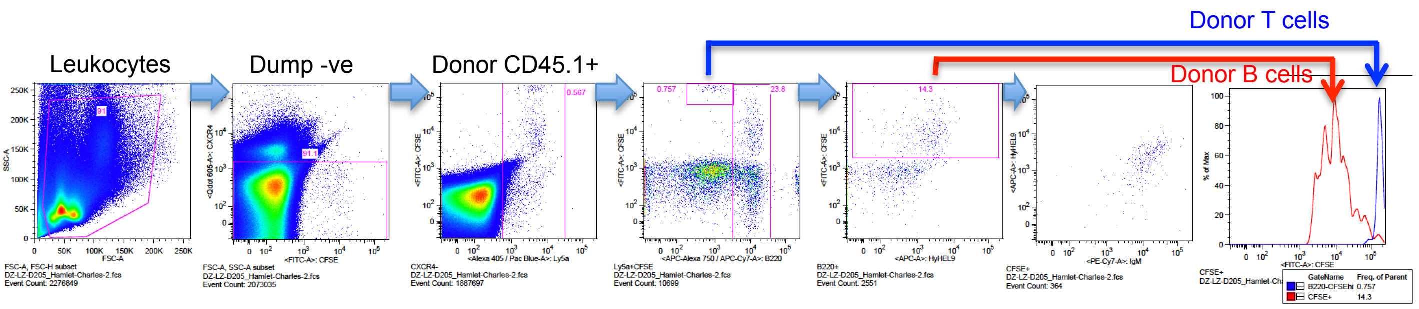

Supplementary Figure 3. Gating strategy for Day 2.5 analysis in Figure 2b-d

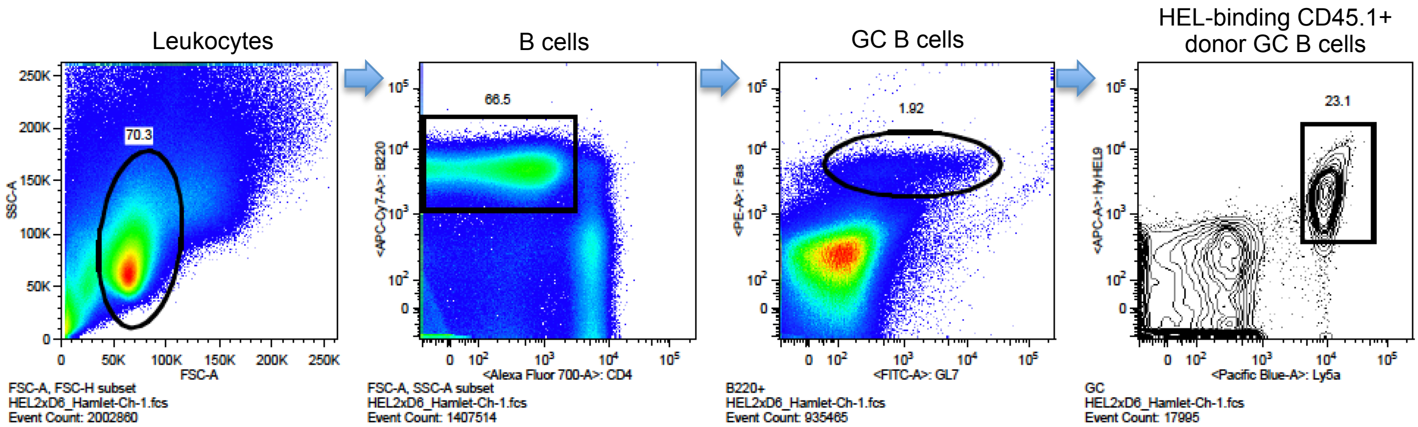

Supplementary Figure 4. Gating strategy for Day 5 analysis in Figure 2e

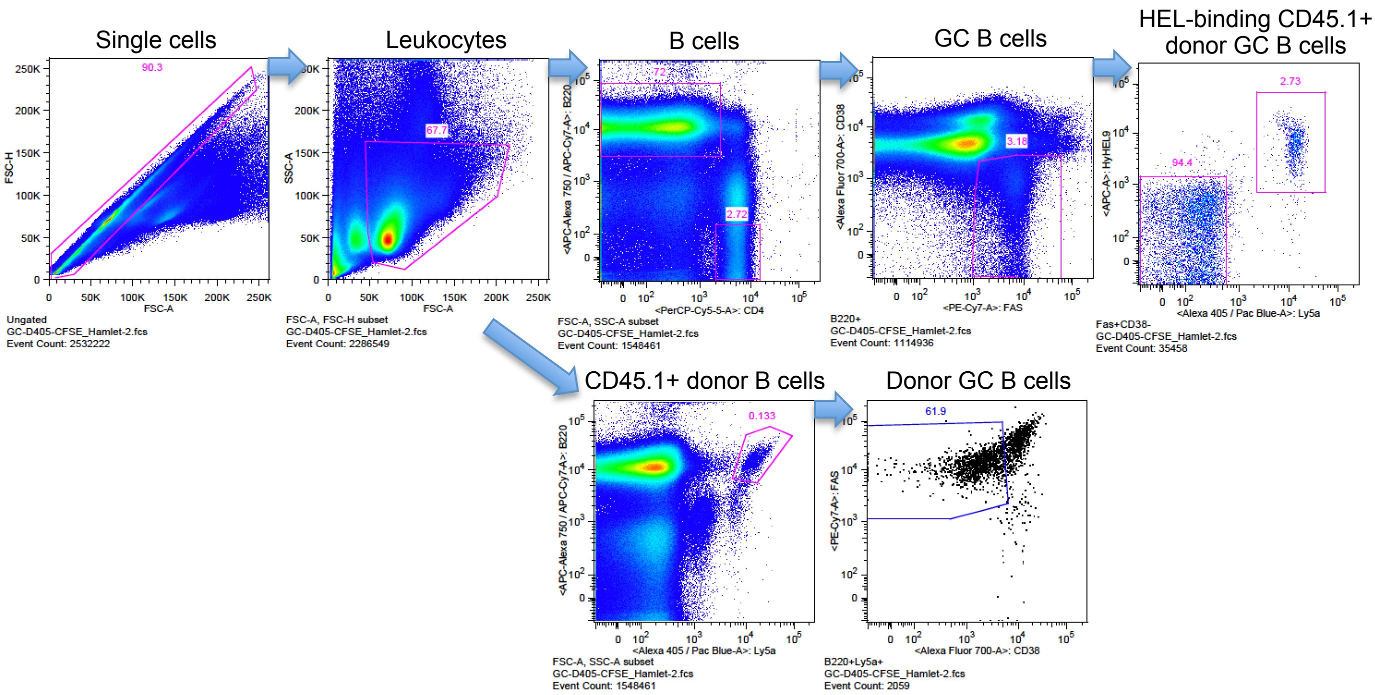

Supplementary Figure 5. Gating strategy for Day 5 analysis in Figure 2e

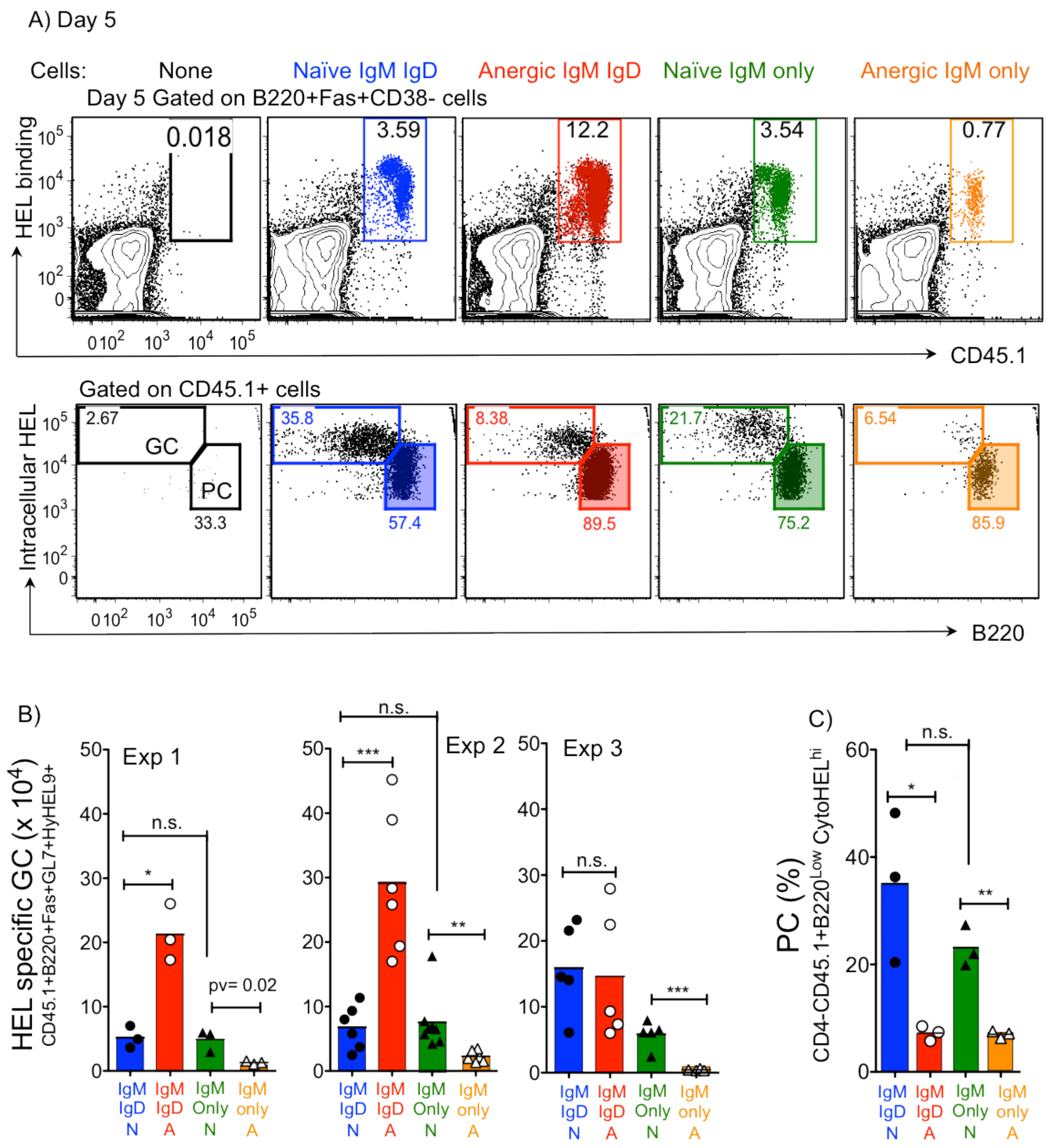

Supplementary Figure 6. Gating used to enumerate HEL-specific GC B cells in Figure 2e

(A) Flow cytometric analysis of spleen lymphocytes from the recipient animals 5 days after immunization, showing the gates applied to measure the percentage of FAS+ CD38- GC B cells (top row). Bottom row is gated on CD45.1+ donor B cells, and measures the percentage differentiated into B220^{low} plasma cells with high intracellular HEL-binding antibody, or into GC B cells. A control group was immunized with mock conjugated SRBCs with no adoptive cell transfer. **(B)** Numbers of FAS+ CD38- HEL binding GC B cells in individual recipients and geometric means for each group from three independent experiments at day 5. **(C)** Percentage of HEL-binding plasma cells in individual recipients and geometric means for each group at day 5. Statistical analysis by ANOVA Bonferroni's Multiple Comparison post-test. (ns=non significant, *p<0.05, **p<0.01, ***p<0.001). Dotted line shows the numbers in the mock group.

Supplementary Figure 6.

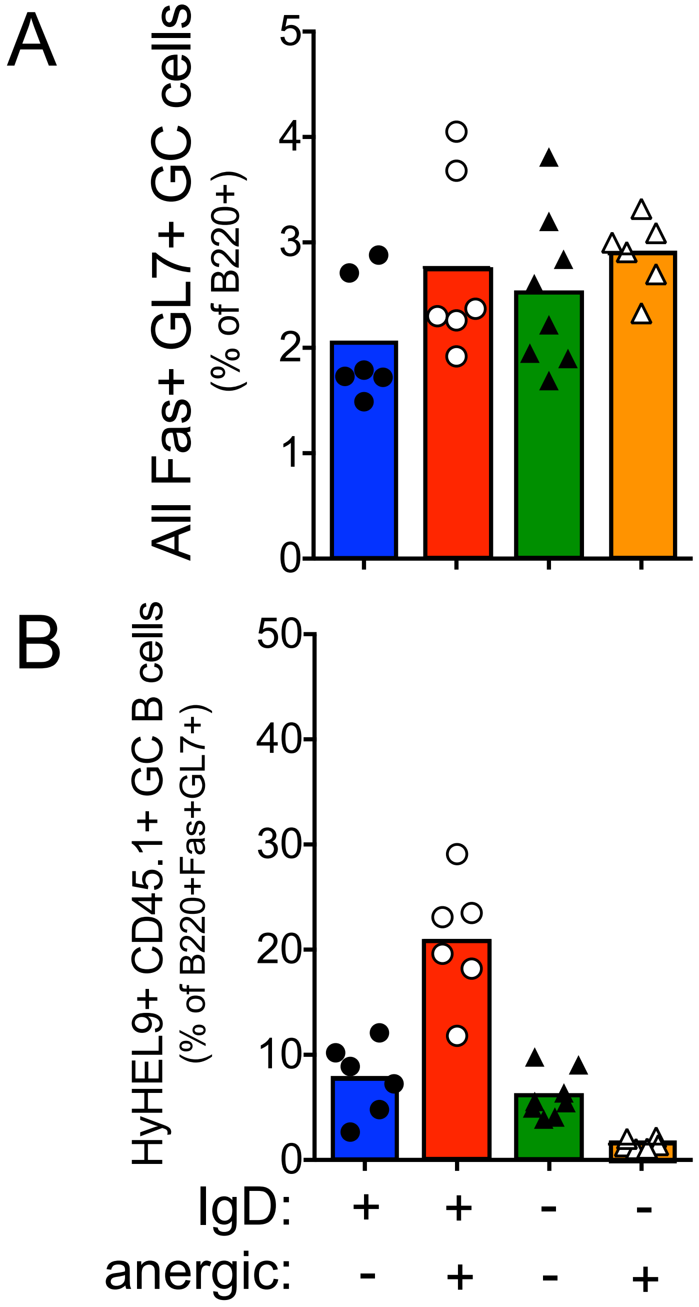

Supplementary Figure 7.

Supplementary data accompanying Fig 2e.

A. The percentage of GC B cells (recipient + donor GC cells) of total B cells in the spleen of the individual recipient animals shown in Figure 1e. Statistical analysis as per Fig 1e, by ANOVA with Bonferroni's Multiple Comparison post test, yielded no evidence for significant differences in the mean between any of the groups (p=0.17).

B. The percentage of HEL-specific CD45.1+ donor-derived GC B cells of all GC B cells in the same recipients. These accounted for a mean of 9% across all 4 groups. Comparison of means between all four groups by ANOVA = p<0.0001.

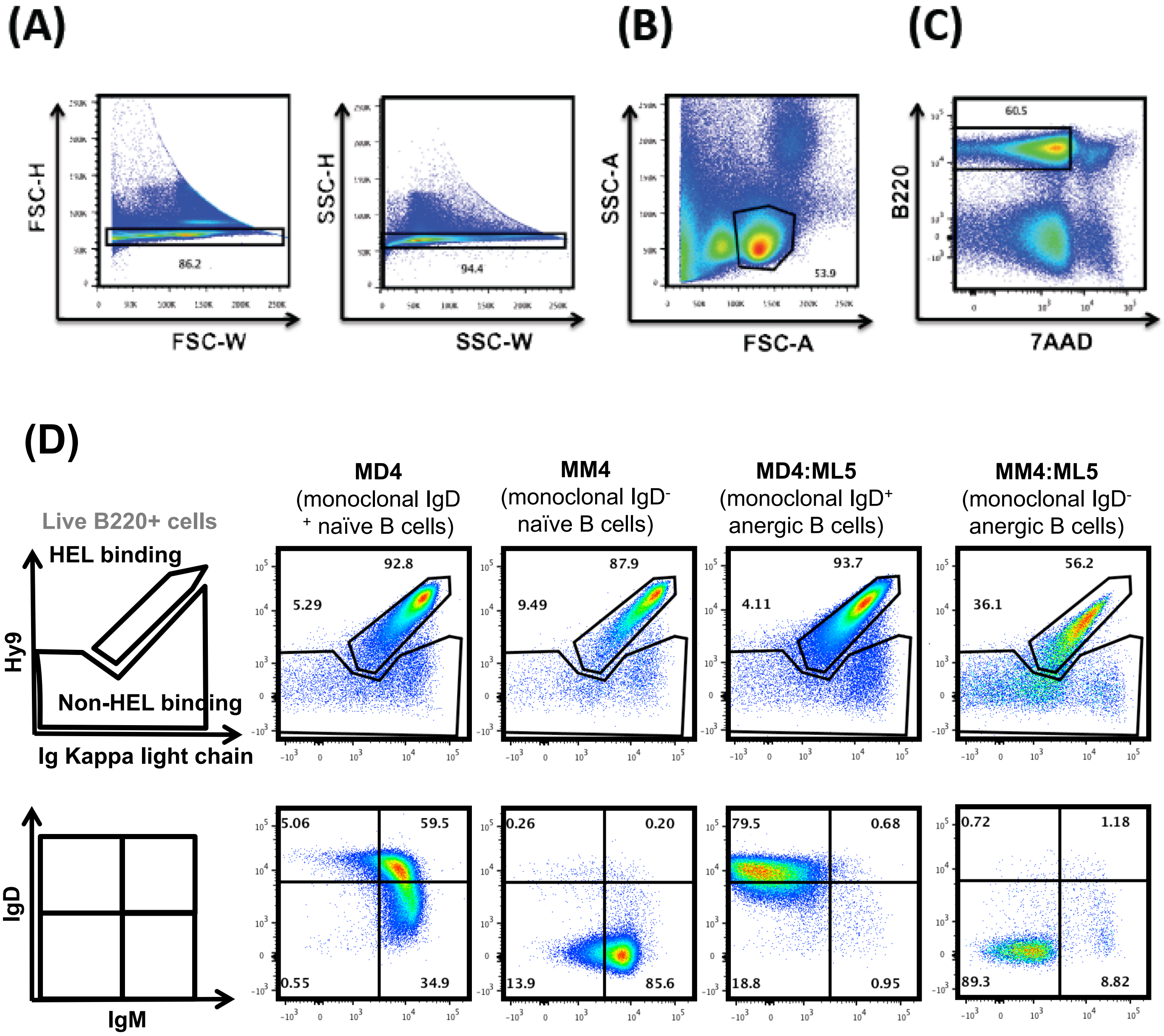

Supplementary Figure 8

Gating strategy used for Figure 2f-h:

(A) Gating on single cells.
(B) Gating on lymphocytes
(C) Gating on viable (live) B cells
(D) Gating on HEL-binding cells, to exclude B cells that have lost expression of the transgene-encoded H or L chain. IgD and IgM staining is shown for information but was not used in gating.

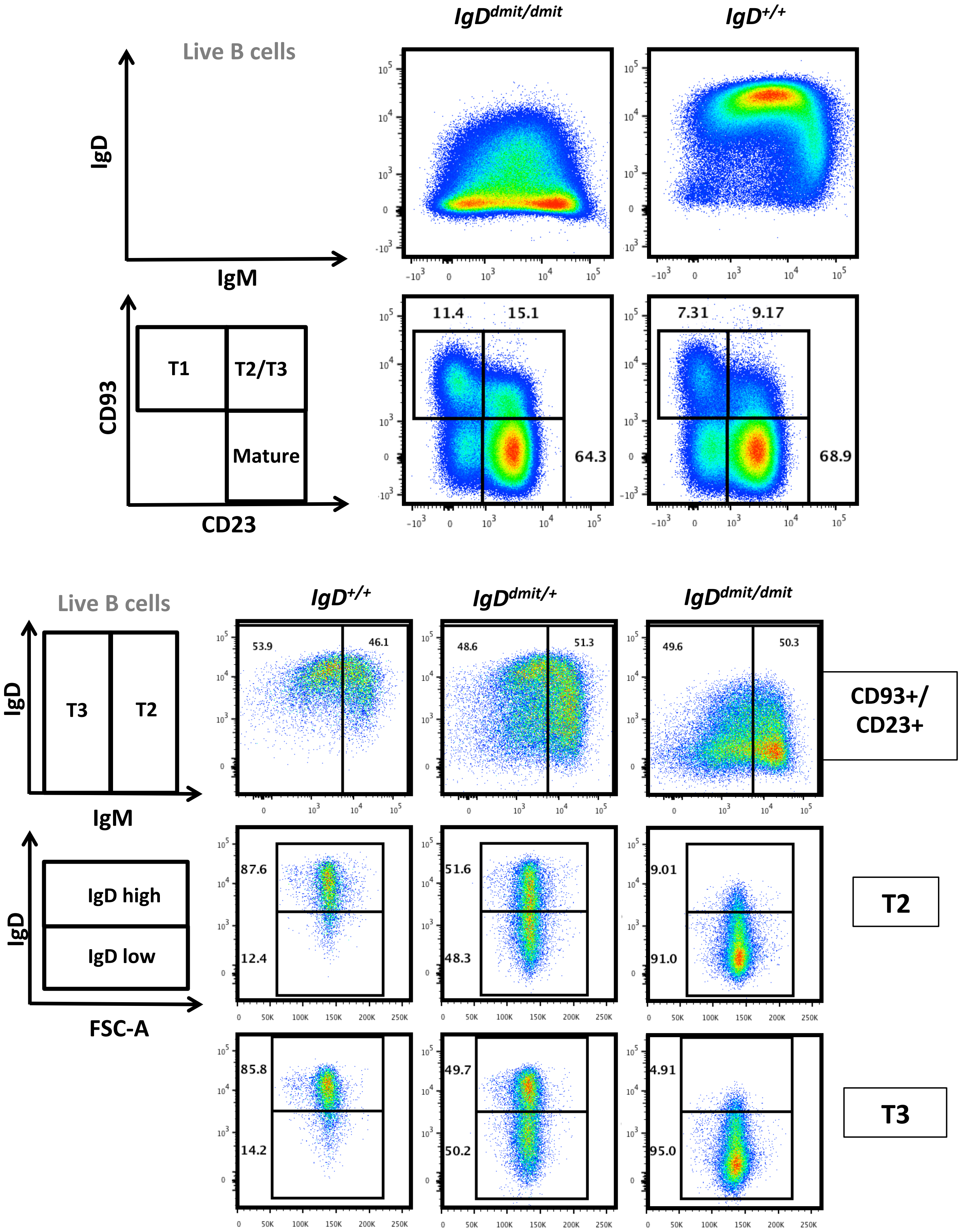

Supplementary Figure 9.
Gating strategy for Fig 3b-d and Figure 6d

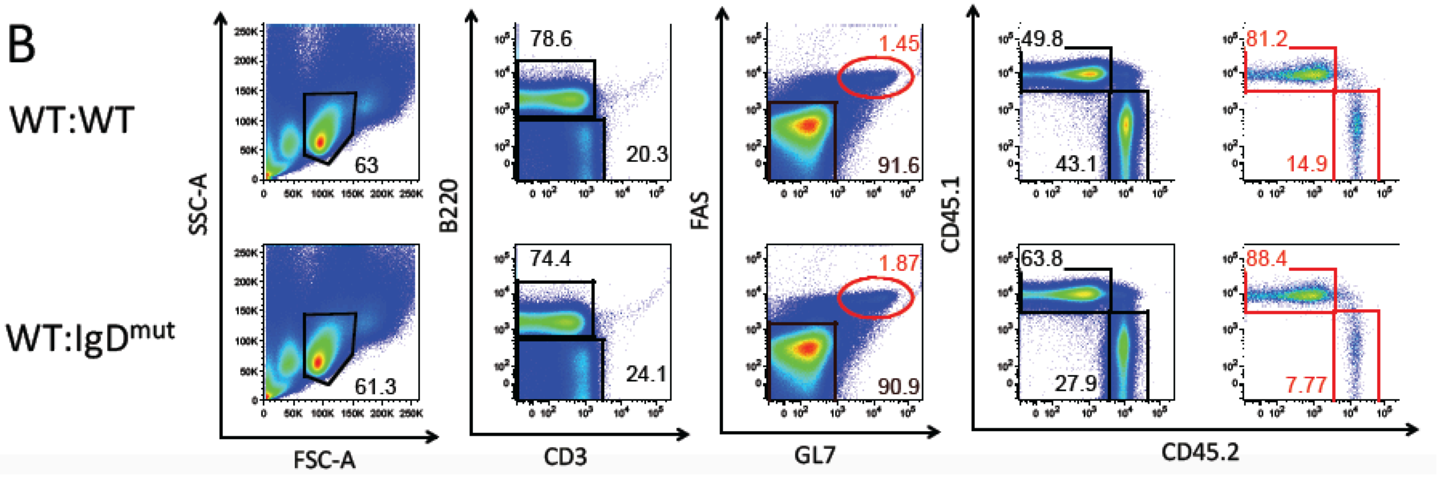

Supplementary Figure 10. Gating strategy to resolve CD45.1 and CD45.2 B cell subsets.

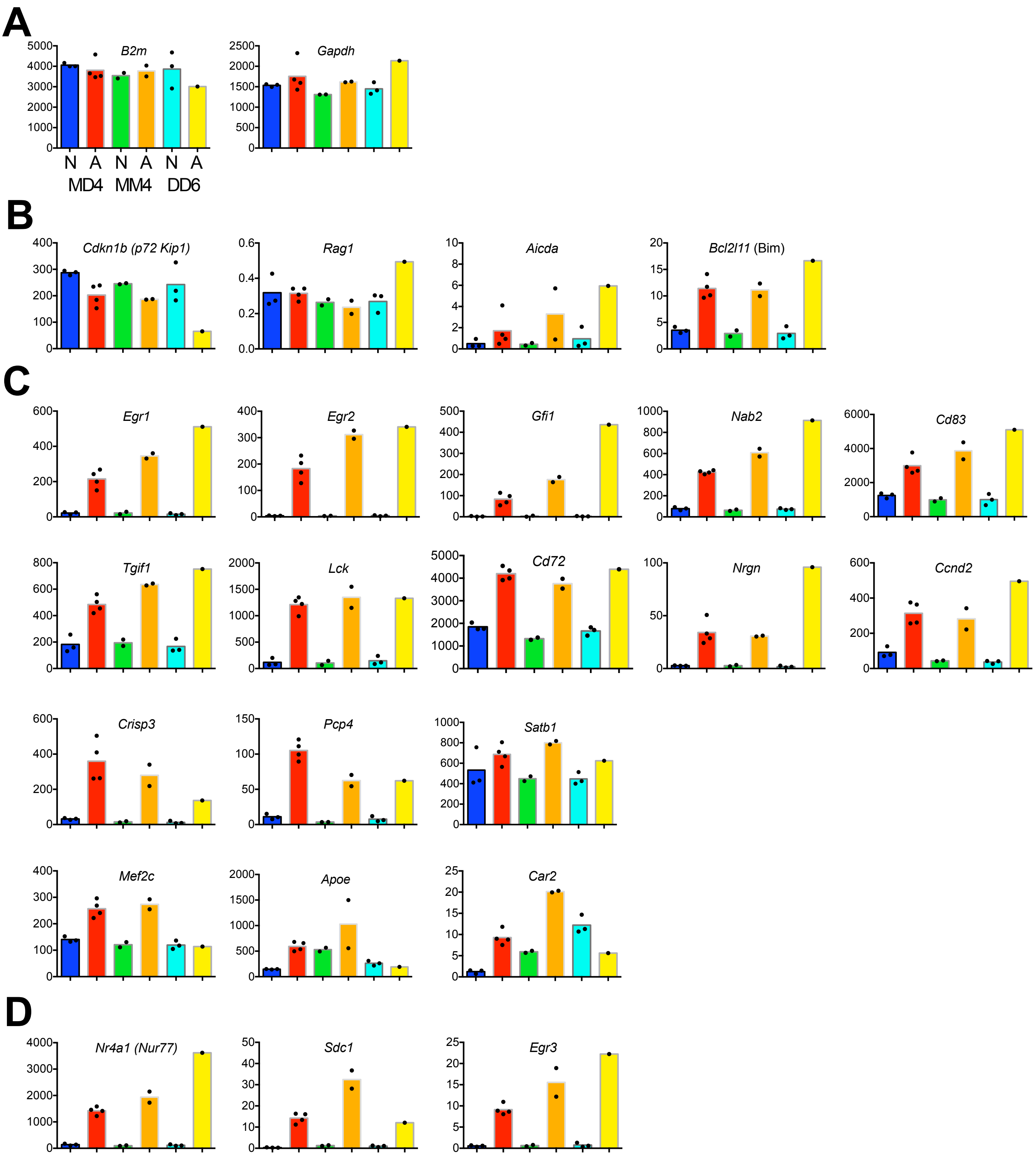

Supplementary Figure 11.

Naïve (N) or anergic (A) HEL-binding CD93-CD23+ mature spleen B cells were sorted from MD4 or MD4:ML5, MM4 or MM4:ML5, and DD6 or DD6:ML5 transgenic mice, and mRNA analysed on Agilent microarrays. Dots show values and columns arithmetic means for indicated mRNA probes from independent mice per genotype except DD6:ML5 where mRNA was pooled from 3 mice. For normalisation, mRNA units are mean pixel intensity of test probe relative to the median intensity of all probes on the same array.

(a) Constitutively expressed genes.

(b) Genes previously identified as induced when mature B cells lose tonic BCR signals.

(c) Genes previously identified as being induced in anergic MD4:ML5 B cells compared to naïve MD4 B cells using first-generation Affymetrix arrays.

(d) Other previously identified anergy-induced genes.

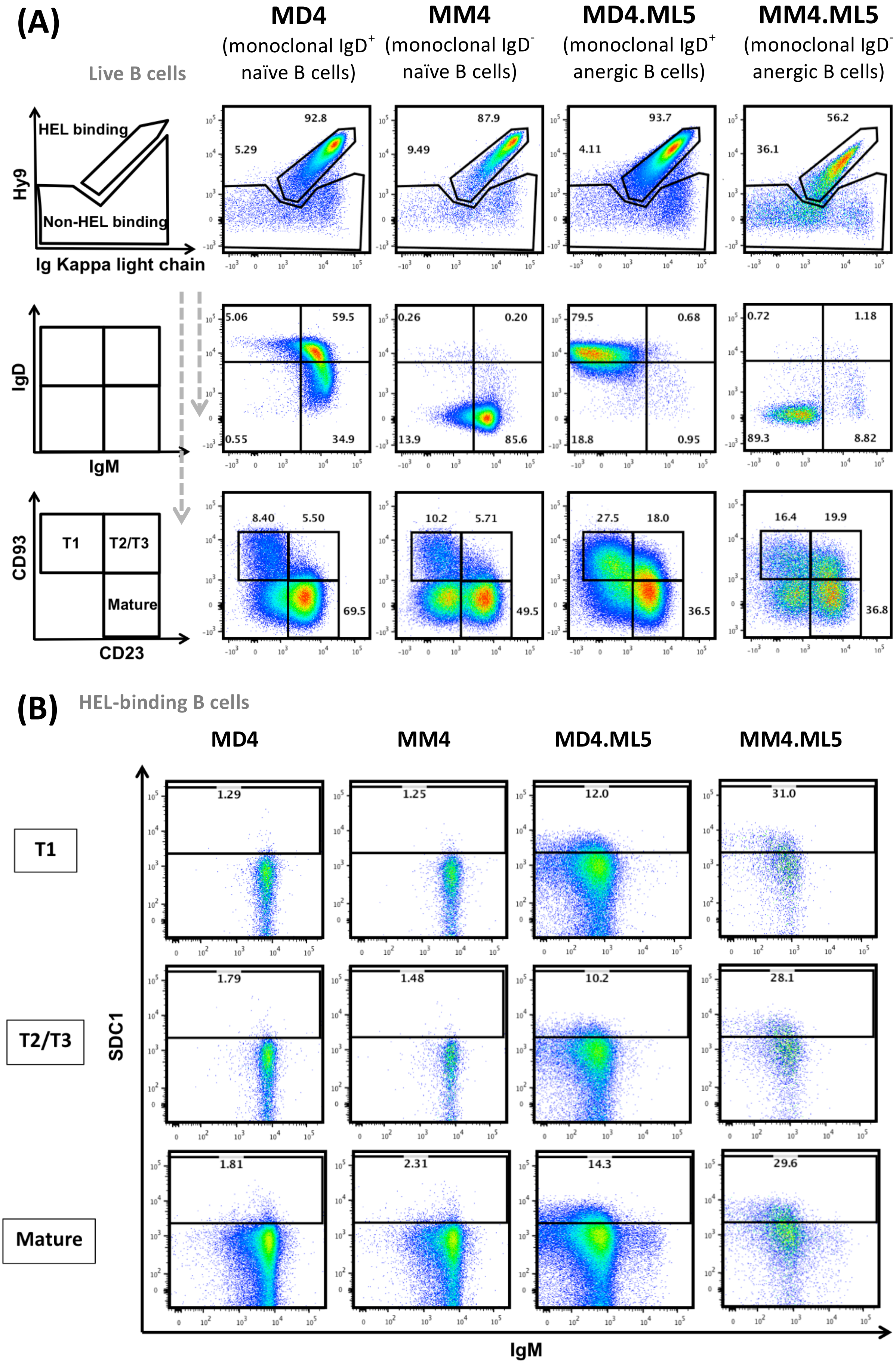

Supplementary Figure 12.

Gating strategy for Figure 5a, b, d

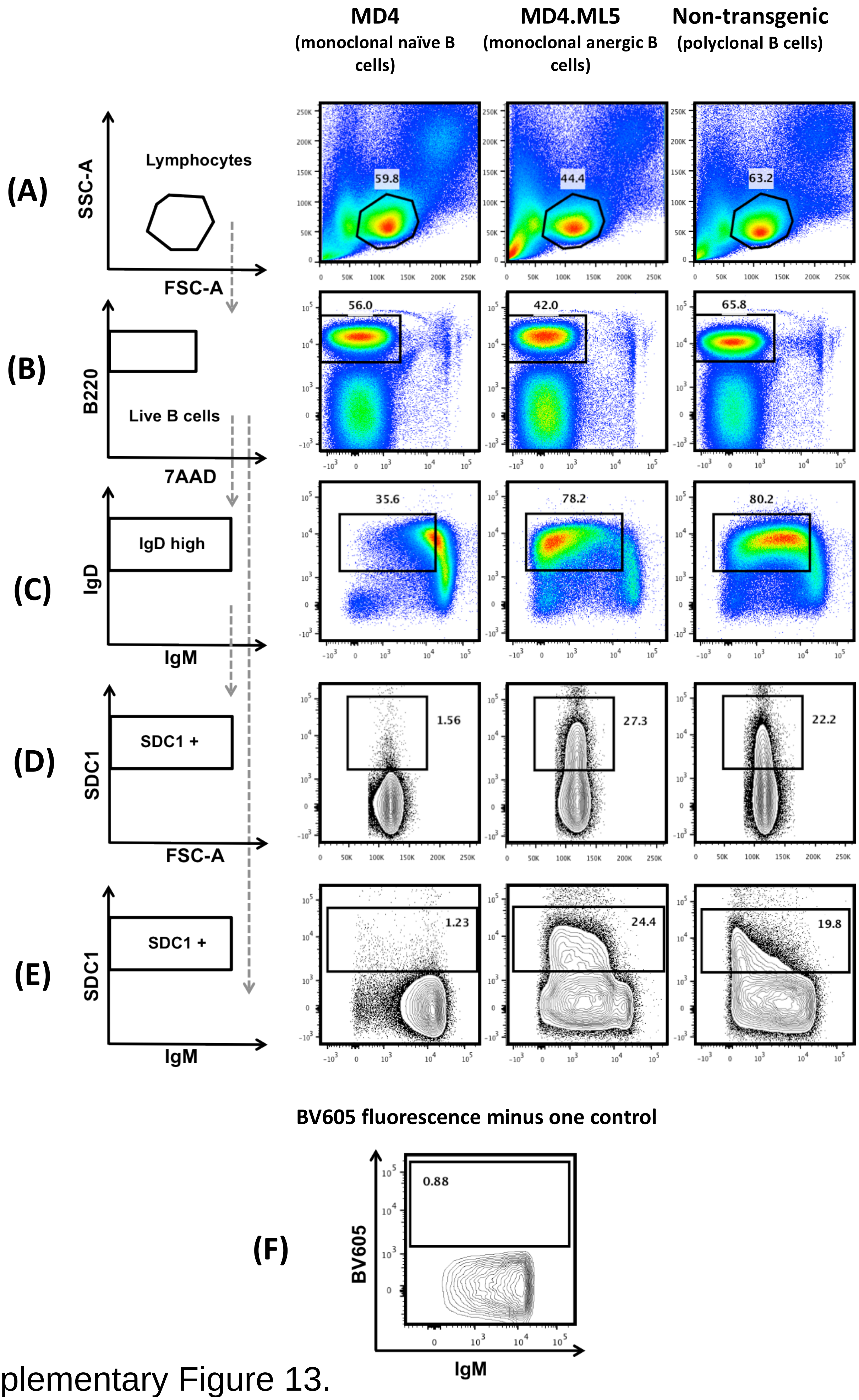

Supplementary Figure 13.

Gating strategy for Figure 5c

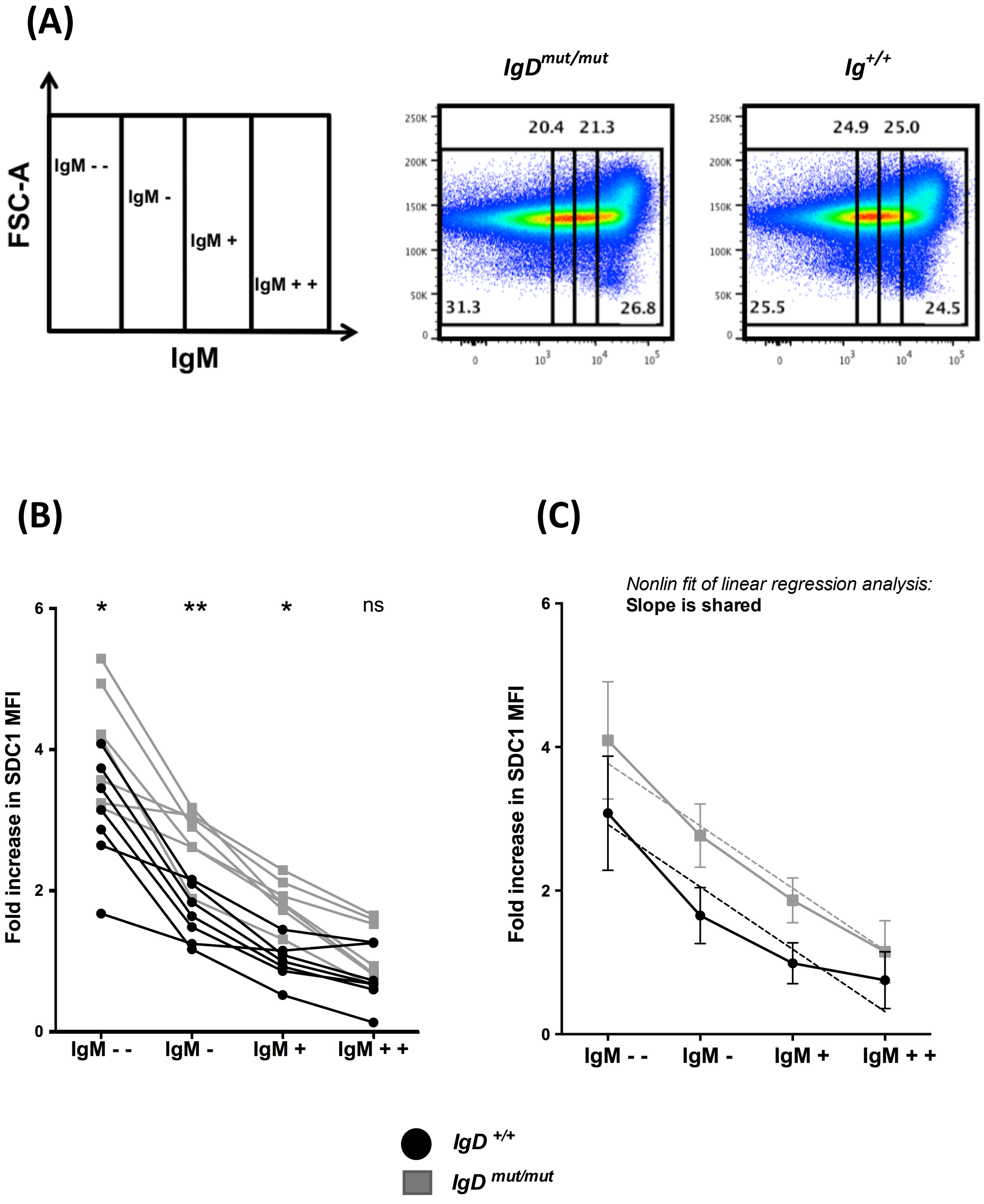

Supplementary Figure 14.

Gating strategy for Figure 6c.
