# Supplementary material for: IgD attenuates the IgM-induced anergy response in transitional and mature B cells
Source: Nat Commun. 2016 Nov 10;7:13381. doi: 10.1038/ncomms13381 (PMC5109548; doi:10.1038/ncomms13381)
Supplement: Peer Review File [file ncomms13381-s4.pdf]

Transferred manuscripts:
Editorial Note: this manuscript has been previously reviewed at another journal that is not operating a transparent peer review scheme. This document only contains reviewer comments and rebuttal letters for versions considered at Nature Communications.

REVIEWERS' COMMENTS:

Reviewer #1 (Remarks to the Author):

Criticisms were addressed.

Reviewer #2 (Remarks to the Author):

The authors have satisfactorily addressed the concerns raised in the initial review.
